# *Cynara cardunculus* L. as a Multipurpose Crop for Plant Secondary Metabolites Production in Marginal Stressed Lands

**DOI:** 10.3389/fpls.2020.00240

**Published:** 2020-03-31

**Authors:** Helena Domenica Pappalardo, Valeria Toscano, Giuseppe Diego Puglia, Claudia Genovese, Salvatore Antonino Raccuia

**Affiliations:** Consiglio Nazionale delle Ricerche-Istituto per i Sistemi Agricoli e Forestali del Mediterraneo, Catania, Italy

**Keywords:** heavy metals, salt, sprout, gene expression, antioxidant activity

## Abstract

Cardoon (*Cynara cardunculus* L.) is a Mediterranean crop, member of the Asteraceae family, characterized by high production of biomass and secondary metabolites and by a good adaptation to climate change, usable in green chemistry, nutraceutical, and pharmaceutical sectors. Recent studies demonstrated the ability of cardoon to grow up in a stressful environment, which is associated with enhanced biosynthesis of biologically active compounds in these plants, and this effect is increased by abiotic stresses (salt, heat, pollution, and drought stress) that characterize many world marginal areas, affected by the climate changes. The plant response to these stresses consists in implementing different processes that modify some plant biological functions, such as alleviating both cellular hyperosmolarity and ion disequilibrium or synthesizing antioxidant molecules. The aim of this work was to investigate different cardoon response mechanisms to abiotic stresses and to evaluate their influence on the biologically active compounds biosynthesis. Following this purpose, we analyzed the ability of cardoon seeds to germinate under different salt stress conditions, and on the sprouts obtained, we measured the total phenol content and the antioxidant activity. Moreover, the growth of cardoon seedlings grown under heavy metals stress conditions was monitored, and the expression levels of heavy metal transport–associated genes were analyzed. The results showed the ability of cardoon plants to tolerate abiotic stress, thanks to different defense mechanisms and the possibility to obtain biomass with high content of biologically active molecules by exploiting the natural tolerance of this species for abiotic stresses. Moreover, we identified some important genes encoding for metal transportation that may be involved in arsenic and cadmium uptake and translocation in *C. cardunculus*. Then, this species can be considered as a promising crop for green chemistry and energy in marginal lands.

## Introduction

The climate, over the centuries, has always changed because of natural processes, but in the last 100 years, these changes have been much more severe and much faster than the changes that occurred in the past.

Climate change caused an increase in unfavorable or stressful environment. Abiotic stresses, such as drought, heat, cold, salt, or heavy metals like arsenic (As) and cadmium (Cd) in the soil, are exacerbated by climate change (Fedoroff et al., 2010). Drought and salinity are prime environmental stresses that influence the geographical distribution of plants in nature, limit their agriculture productivity, and threaten food security (Zhu, 2016). More than 6% of the world’s total land and approximately 20% of irrigated land are affected by salt stress (Munns and Tester, 2008).

Human activities, industrialization, and modern agricultural practices are mainly responsible for the increase in environmental contamination by heavy metals (Kavamura and Esposito, 2010; Miransari, 2011; Singh et al., 2016). The use of pesticides, fertilizers, municipal and compost wastes, and also heavy metal release from smelting industries and metalliferous mines contributed to contaminate, in a decisive way, large areas of land with heavy metals (Yang et al., 2005; Singh et al., 2016). Either the presence of salt or heavy metals in soils affect the biological cycle of the plants. Although salt stress influences all growth stages of a plant, seed germination and seedling growth stages are known to be more sensitive for most plant species (Begum et al., 1992; Cuartero et al., 2006), and germination has been reported to decline with increasing salinity levels (Houle et al., 2001). Instead, the presence of heavy metals during seedling growth and plant establishment stage causes morphological abnormalities leading to yield reduction (Amari et al., 2017). Both these stresses give rise to the production of reactive oxygen species (ROS) compounds, such as O_2_, H_2_O_2_, and OH^–^ (Mittler, 2002), which damage membranes and macromolecules. The plants, as a response to these stresses, have developed several strategies. One of these is the accumulation of compatible solutes in their organs in response to osmotic stress; the primary function of these solutes is to maintain cell turgor and thus the driving gradient for water uptake (Gupta and Goyal, 2017). Another strategy is the production of antioxidant compounds (ROS scavengers), such as polyphenols, which improve the antioxidant defense and can thus increase tolerance to different stress factors (Cushman and Bohnert, 2000).

The most commonly found heavy metals at contaminated sites are arsenic and Cd (Mulligan et al., 2001). These elements are non-essential metals with no known biological function in plants, so there is not a specific transporter system for them, but they can use the same transporters of the essential nutrient uptake (Verbruggen et al., 2009; Mendoza-Cózatl et al., 2011). Different gene families were proposed to be putatively involved in the uptake of As and Cd in plants (Fan et al., 2018). The Zinc Iron Protein (ZIP) family was mainly associated with the metal transportation from the soil (Guerinot, 2000). ZIP5 and ZIP6 obtained from *Thlaspi caerulescens* cloned in *Arabidopsis thaliana* indicated that both genes act in metal homeostasis (Wu et al., 2009). Natural Resistance of Macrophage (NRAMP) are metal transporters, and in *A. thaliana*, a non-hyperaccumulator plant, NRAMPs have high affinity with Fe and Mn transporters (Thomine et al., 2003; Lanquar et al., 2010) and also retain heavy metal transport (Ni and Cd) ability (Thomine et al., 2003; Oomen et al., 2009). In rice, *OsNRAMP1* expression is induced during As stress at the same time of other stress-responsive genes, transporters, heat shock proteins, metallothioneins, and sulfate-metabolizing proteins (Tiwari et al., 2014). NRAMP3 and NRAMP4 are responsible for Cd2^+^ efflux from the vacuole (Verbruggen et al., 2009). The heavy metal ATPases (HMAs) operate in heavy metal transport and play a role in metal homeostasis and tolerance (Gupta et al., 2013). The HMA3 was proposed to be involved in the vacuolar storage of Cd in *A. thaliana* (Verbruggen et al., 2009). Among the phosphate transporters (PHT) family, in *Arabidopsi*s, overexpression of PHT1 or PHT7 causes hypersensitivity to arsenate, due to increased As uptake, whereas As resistance is enhanced through YCF1-mediated vacuolar sequestration (LeBlanc et al., 2013). The large ATP-binding cassette (ABC) transporters are involved in the transfer of different substances, including carbohydrates, lipids, xenobiotics, ions, and heavy metals (Kim et al., 2007). The *AtABCC1* and *AtABCC2* are the major vacuolar transporters of peptide-chelating heavy metals (Song et al., 2010) mediating AsIII–PC complex transport to the vacuole in *Arabidopsis*.

The possibility of using some species for phytoremediation of soils has already been widely studied, but the identified hyperaccumulators are mainly herbaceous plants, which have some limits: metal selectivity, low biomass, shallow root systems, and slow growth rates (Krämer, 2010; Cun et al., 2014; Fan et al., 2018). Therefore, some high-biomass perennial plants have been studied recently as potential candidates for phytoremediation (Fan et al., 2018). Among these crops, *Cynara cardunculus* L. plays an increasingly important role; previous research (Llugany et al., 2012), in fact, demonstrated both the ability of this species to survive quite well in polluted and stressed soil and its beneficial properties linked to its nutritional and nutraceutical characteristics to protect the body from oxidative stress.

Cardoon (*C. cardunculus* L.) is a perennial species of the *Asteraceae* family with annual growth cycle. The plant is well adapted to Mediterranean climates characterized by hot and dry summers (Raccuia and Melilli, 2007; Toscano et al., 2016). It comprises three taxa, *C. cardunculus* L. subsp. *scolymus* (L.) Hegi = *C. cardunculus* L. var. *scolymus* (L.) Hayek (globe artichoke), *C. cardunculus* L. var. *altilis* DC. (leafy or domestic cardoon), and *C. cardunculus* L. var. *sylvestris* Lam. (wild cardoon), considered to be the wild ancestor of globe artichoke (Rottenberg and Zohary, 1996; Raccuia et al., 2004b). The wild cardoon is a robust thistle well-adapted to Mediterranean semiarid climate. The domestic one is well known for its high biomass and for its use as raw material in green chemistry. In fact, from this plant, it is possible to realize an innovative range of bioproducts (bioplastics, biolubricants, home and personal care items, food fragrances, plant protection additives, etc.), with a positive impact on the environment and farmers’ income (Toscano et al., 2016). Moreover, the cardoon biomass, which contains cellulose, hemicellulose, and lignin, can be used to produce energy (Raccuia and Melilli, 2007; Ierna et al., 2012; Ciancolini et al., 2013; Toscano et al., 2016; Ottaiano et al., 2017; Gominho et al., 2018; Petropoulos et al., 2019; Turco et al., 2019). Recent studies, on morphological and physiological characteristics and on seed germination process, showed intraspecific variability among different cardoon populations under salt and moisture stresses (Mauromicale and Ierna, 2004; Raccuia et al., 2004a; Benlloch-González et al., 2005; Pagnotta and Noorani, 2014; Toscano et al., 2016). The resistance developed by this plant to both salt and heavy metal stresses was shown to be associated with a greater synthesis and the accumulation of the secondary metabolites (Mauromicale and Licandro, 2002; Raccuia et al., 2004b; Argento et al., 2016; Leonardi et al., 2016; Pappalardo et al., 2016), consisting of high amounts of polyphenolic compounds and inulin conferring healthy properties to this plant (Raccuia et al., 2004a; Pandino et al., 2011; Genovese et al., 2016a, b).

The aim of this work was to investigate the cardoon response mechanisms to different abiotic stresses. To this end, we focused on the ability of the seed to germinate under saline stress and its effect on the synthesis of antioxidant compounds. Moreover, we monitored the seedling growth in presence of heavy metals (As and Cd) and the expression levels of ion transporter genes associated with their translocation.

## Materials and Methods

### Plant Materials

For the different trials of this study, two genotypes were used: a wild cardoon genotype, “A14SR” (*C. cardunculus* L. var. *sylvestris*), and one domestic cardoon variety (*C. cardunculus* L. var. *altilis*). All the genotypes belong to the *cardoon* germplasm of the section of Catania of the National Research Council–Institute for Agricultural and Forest System in the Mediterranean (CNR-ISAFOM of Catania, Italy). The seeds were collected by hand from dried flower heads by shaking and lightly pounding them, making sure not to damage the seeds themselves during the summertime. The seeds from domestic variety were used to carry out germination tests in salt stress conditions, whereas seeds of both genotypes were used to measure the expression levels of the genes associated with salt tolerance and heavy metal transport.

### Germination Test With NaCl

To verify the effect of salt stress on the biosynthesis and the concentration of secondary metabolites, stress conditions were induced during the germination of domestic cardoon seeds by three different salt concentrations: 0, 60, and 120 mM of NaCl.

**FIGURE 1:**
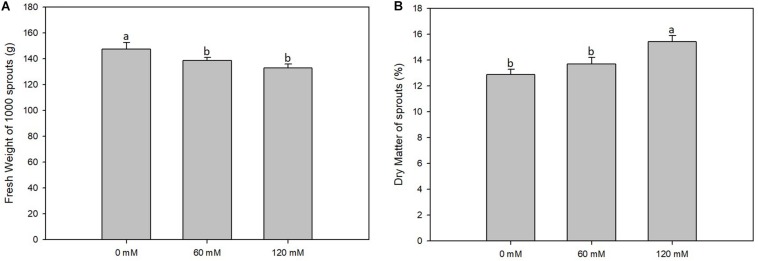
Fresh weight **(A)** and dry weight **(B)** percentage of *altilis* sprouts grown at different salt concentrations (0, 60, and 120 mM). The error bars represent the error mean of three biological replicates. Different letters indicate differences at *P* ≤ 0.05 (ANOVA).

For this experiment, 10-cm diameter Petri dishes were used with a filter paper placed at the bottom. Three experimental replicas were prepared, consisting of eight Petri dishes each for every salt concentration (0, 60, and 120 mM). All dishes were filled with 30 seeds and 10 mL of saline solution; then, each plate was closed with parafilm and placed in the growth chamber with a 12-/12-h light-dark cycle photoperiod and a 15°C/25°C temperature cycle (Figure 1) (Raccuia et al., 2004a). The young sprouts obtained were grown for 20 days.

### Total Phenol Content and Antioxidant Activity

After 20 days of growth, fresh and dry weight of 1,000 sprouts was recorded, and all the sprouts were stored at −80°C to be used for TPC (total phenol content) and the AA (antioxidant activity) measurements.

Phenolic compounds were extracted from the plant material with 80% methanol solution with a ratio of 1:10 (wt/vol); the mixture thus obtained was sonicated for 44 min in an ultrasonic tank. Afterward, the sample was centrifuged at 3000 *g* for 3 min, and the supernatant obtained filtered with a 0.20-μm filter. The obtained sample was used for the determination of TPC and AA.

The TPC was determined by the Folin–Ciocalteu method, as described by Dewanto et al. (2002). The results were expressed as milligrams of gallic acid equivalent (GAE)/g of sample. The calibration curve of gallic acid ranged from 25 to 200 μg/mL.

The AA was determined on cardoon sprouts using 2,2 diphenyl-1-picrylhydrazyl (DPPH) radical according to Brand-Williams et al. (1995). The antioxidant capacity was calculated by using a calibration curve obtained from known concentrations of Trolox in MeOH (10–200 μmol/L). The results were expressed as micromoles of Trolox equivalent (TE)/g of sample. All samples were analyzed in triplicates.

### Seedlings Growth Analysis in Heavy Metal Stress Conditions

For this experiment, *altilis* and *sylvestris* seeds were sown in one-half MS medium with the addition of 0, 25, or 50 μM of cadmium sulfate hydrate and sodium arsenate dibasic heptahydrate, at 20°C/25°C and 12-h photoperiod. After 21 days from the seed sowing, the seedlings were collected and dissected into shoot and root portions measuring the length of the two organs. Shoot height was measured from root collar to the longest leaf extremity, and root length was measured from the root collar to the longest root apex.

### Identification of Cardoon Genes Likely Associated With Plant Stress Response

To identify the cardoon orthologous genes involved in plant stress response, up to 100 mg of tissue was used to extract total RNA using protocols based on Chang et al. (1993). One microgram of total RNA, for each biological replicate, was reverse transcribed using ImProm-II^TM^ Reverse Transcription System (Promega, Madison, WI, United States) according to the manufacturer’s instructions.

To isolate cardoon genes involved in heavy metal uptake, we selected the NRAMP3, ZIP11, ABCC, HMA, and PHT *A. thaliana* genes sequences, and we searched orthologous sequences within the *C. cardunculus* var. *scolymus* genome v.1^1^ and cardoon transcriptome (Puglia et al., 2019) using a local BLASTX and BLASTN analysis, respectively, with an *E*-value cutoff of 10^–5^ for both algorithms. The same procedure was carried out for the isolation of cardoon housekeeping genes, EF1 alpha, and GAPDH, which were used in the reverse transcriptase–quantitative polymerase chain reaction (RT-qPCR) analyses. The cloning primers were designed within the conserved domain regions using Primer3 software (Rozen and Skaletsky, 1999) (Table 1). The PCR was performed with PerfectTaq DNA polymerase (5 PRIME, Hilden, Germany), according to the manufacturer’s instructions and cloned into the pJET vector (CloneJET PCR Cloning Kit; Thermo Scientific, Waltham, MA, USA). The DNA sequences were deposited to GenBank database (GenBank accession numbers from MN889990 to MN889996). To confirm the isolation of partial coding sequence of the genes, the obtained sequences were searched over the nucleotide collection (nt) database with BLASTN algorithm, and the first match was considered to confirm the correctness of isolated sequence. Moreover, to identify the protein region domain within the obtained contig sequences, we carried out a BLASTP analysis of the translate sequences choosing nr (non-redundant protein sequences) database excluding *C. cardunculus* species (taxid 4265) or limited to *C. cardunculus* species (taxid 4265).

**TABLE 1:** Cloning and RT-qPCR primer sequences designed for cardoon genes likely associated with heavy metal uptake and for housekeeping genes.

Gene name	Cloning forward primer sequence	Cloning reverse primer sequence	T_*a*_	RT-qPCR forward primer sequence	RT-qPCR reverse primer sequence	Tm
*NRAMP3*	TTGATGCTACAATCAACCGA	GGGCAGAGTAGTACCATCAC	57°C	GGTGTAAGGAAGTTAGAGGCCC	AGCTTTGGAACCACGAGACC	60°C
*ZIP 11*	GAGATTGTCCATAGAGCTTTCCA	CCATGCCTCGTTTCCTTTTCT	48°C	TGCCTCGTTTCCTTTTCTTC	CTCGGGTGCTTCGTCGT	60°C
*HMA*	CGCACAGTCTCGTTCAGTTG	GGTTCCCACGTCCGCAAG	50°C	CGGGCACGATTACTAGAGGA	CTAGCCTTGCTCTCGATGCT	60°C
*ABCC*	TGACCCCTTCAATGAGCACA	CGGTCACAATCGATGATGGT	54°C	TAAGTCTTTCGCGTGCATTG	TTCTTCGCGAATGGTCTTTT	60°C
*PHT*	GCATCTTCCTCATTCTCGCC	GTCATGGCCACCCTTTGTTT	48°C	AAGATTTCAGCAGGGACGAC	ACGACAACCGTCTTGGATTC	60°C
*GAPDH*	TGARTCHACYGGTGTCTTCA	TCRAYVACACGRGARCTGTA	53°C	AGTACGACAGTGTTCATGGCC	CTGAAGCCGAAAACAGCGAC	60°C
*EF*	TCCTTCTTGTCCACGCTCTT	AGTTGGCCGTGTTGAAACTG	53°C	TGACCCCAGTTTCAACACGG	AAGAGGCCATCAGACAAGCC	60°C

### Transcriptional Levels of the Genes Likely Associated With Heavy Metal Stress Response

RNA was extracted from shoots and roots of plants grown for 2 and 3 weeks in presence of different concentrations of Cd or As. Reverse transcription reactions were performed by using ImProm-II^TM^ Reverse Transcription System (Promega) according to the manufacturer’s instructions. One microgram of total RNA was used for the cDNA synthesis. The RT-qPCR primers for all the heavy metal–associated cardoon genes were designed using primer3 website (Rozen and Skaletsky, 1999), setting annealing temperature at 60°C, and the gene expression levels were normalized using isolated housekeeping genes. The real-time PCR reactions were performed on a Rotorgene 6000 cycler (Qiagen, Hilden, Germany) with the QuantiNova SYBR Green Kit (Qiagen). At least three biological and three technical replicates per biological replicate were analyzed using real-time PCR analysis.

### Data Analysis

All data were submitted to Bartlett test for the homogeneity of variance and then analyzed using analysis of variance (ANOVA) with CoStat program (CoHort Software, Monterey, CA, United States). Means were separated on the basis of the least significant difference, when the *F* test was significant at least at 0.05 probability level. Analysis of variance at three ways completely randomized was used to analyze the factors that mostly influenced the transcriptional levels of the considered genes, whereas ANOVA at one way was used to highlight the factors’ influence on the variable analyzed. In order to provide a more comprehensive analysis of the effect of the individual metal stress treatments, we carried out a principal component analysis (PCA) using the RT-qPCR expression fold-change variation of the five metal-response–associated genes along with the length of shoot and root measured at less than 25 and 50 μM of As and Cd.

## Results

### NaCl Stress

The domestic cardoon germination percentage under NaCl stress was always greater than 87% at all concentrations considered (data not reported).

Fresh weight of 21-day-old sprouts was, on the average of the treatments, 138.1 g. The three different concentrations did not show much difference between them, although the highest weight (147.5 g) recorded for sprouts not subjected to saline treatment was reduced linearly as the salt concentration increased from 60 mM NaCl (138.5 g) to 120 mM (133.0 g) (Figure 1). Dry weight of the shoots showed no significant differences among the tests with different salt concentrations (data not shown). The data obtained led us to deduce that dry matter content found in the sprouts comes essentially from that one contained in the seed and therefore is not affected by salt treatment. This is in accordance with the data obtained concerning the percentage of dry matter, which was affected by salt treatments. In fact, the lowest value was recorded for the control (12.88%), and the highest value was found in the presence of NaCl maximum concentration (15.41%) (Figure 1).

**FIGURE 2:**
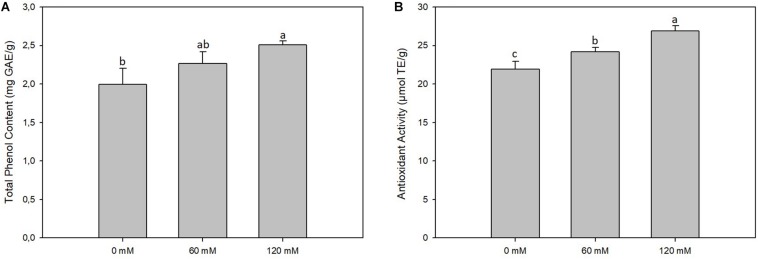
Total phenol content **(A)** and antioxidant activity **(B)** of *altilis* sprouts grown in at different salt concentrations (0, 60, and 120 mM). The error bars represent the error mean of three biological replicates. Different letters indicate differences at *P* ≤ 0.05 (ANOVA).

On the average of the treatments, the TPC in sprouts was 2.26 mg GAE/g, and we observed an increase of up to 25% in sprouts subjected to a higher saline treatment (120 mM) (Figure 2). The AA of sprouts, on the average of the treatments, was 24.96 μmol TE/g, linearly varying between the value of 21.92 μmol TE/g found for the extracts of control shoots and 26.87 μmol TE/g found in the shoots subjected to the higher salt treatment (120 mM) (Figure 2).

### Seedlings Growth Analysis in Heavy Metal Stress Conditions

**FIGURE 3:**
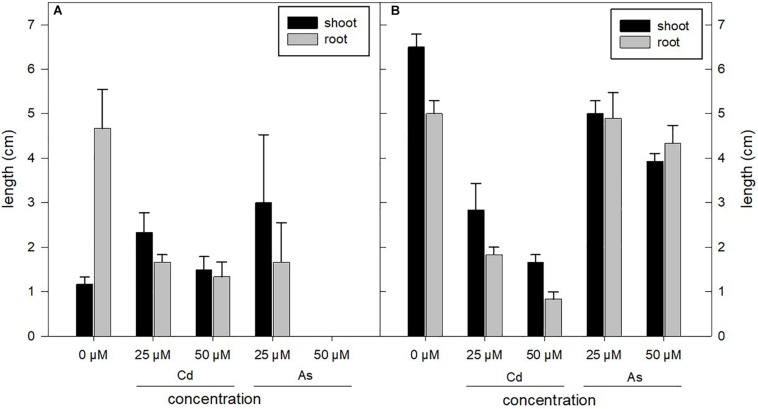
Root and shoot lengths of *sylvestris* **(A)** and *altilis* **(B)** harvested after 21 days of treatment under different concentrations of As and Cd. The error bars indicate the standard deviation of three biological replicates.

Length of seedlings grown under As and Cd stress conditions (0, 25, and 50 μM concentrations) measured after 3 weeks of treatments was differentially affected in the two genotypes analyzed (Figure 3) as the variance analysis confirmed (Table 2). In *sylvestris* genotype, organ–concentration interaction, with 16.97%, was the main cause of variation, differently with respect to *altilis* for which this interaction was not significant. On the contrary, in *altili*s genotype, the metal–concentration interaction was the main and only significant cause of variation (*P* < 0.001), whereas for *sylvestris*, this interaction was not significant.

**TABLE 2:** Analysis of variance of plant length, separated in shoot and root, measured after 21 days of treatment with As and Cd.

Source of variation	*sylvestris*				*altilis*			
	Mean squares				Mean squares			
	*df*	Absolute value		% of total	*df*	Absolute value		% of total
Organ (O)	1	5.84	*	13.94	1	5.14	**	6.83
Metal (M)	1	1.17	ns	2.80	1	30.25	***	40.24
Concentration (C)	2	15.13	***	36.11	2	29.40	***	39.11
O * M	1	0.06	ns	0.15	1	1.14	ns	1.51
O * C	2	16.97	***	40.48	2	1.33	ns	1.77
M * C	2	2.59	ns	6.18	2	7.62	***	10.13
O * M * C	2	0.15	ns	0.35	2	0.31	ns	0.41

Partition of the treatment sum of squares into main effect and interaction. *Significant at 0.05 probability level. **Significant at 0.01 probability level. ***Significant at 0.001 probability level. ns, non-significant.

Under As treatment, *altilis* genotype presented a growth rate similar to the control (0 μM), whereas under Cd treatment, a significant reduction in roots and shoots length was observed, showing a lower tolerance to the metal than that observed in the previous germination phase (Figure 3). However, shoots remained vital with no evidence of chlorosis. In *sylvestris* subjected to both metal stresses, root and shoot lengths decreased with the increase in heavy metal concentration, but at 25 μM, this was more evident in Cd than in As. Root length resulted more than twofold lower compared to untreated controls.

The root/seedling length ratio was also influenced by the metal and concentration used. In particular, for *sylvestris* in presence of Cd, the ratios were 80, 42, and 47% at 0, 25, and 50 μM, respectively, whereas they were 43, 39, and 33% in *altilis* at the same metal concentrations. As for As treatments, the ratios were 80 and 35% at 0 and 25 μM in *sylvestris*, and at 50 μM, seeds germinated, but their growth was arrested eventually. For *altilis* at 0, 25, and 50 μM of As, the root/seedling length ratios were 43, 49, and 52%, respectively, showing minor changes compared to control. Regarding the shoot/roots length ratio across the treatments, it varied very strongly in *sylvestris*, compared to both *altilis* and control.

### Identification of Cardoon Genes Likely Associated With Plant Stress Response

The BLASTN and BLASTP analyses (Supplementary Tables S1, S2) of obtained cDNA contigs confirmed the identity and reliability of our sequences, not previously identified in cardoon. In fact, the nucleotide sequences present in cardoon database are just predicted from amino acid sequences.

**FIGURE 4:**
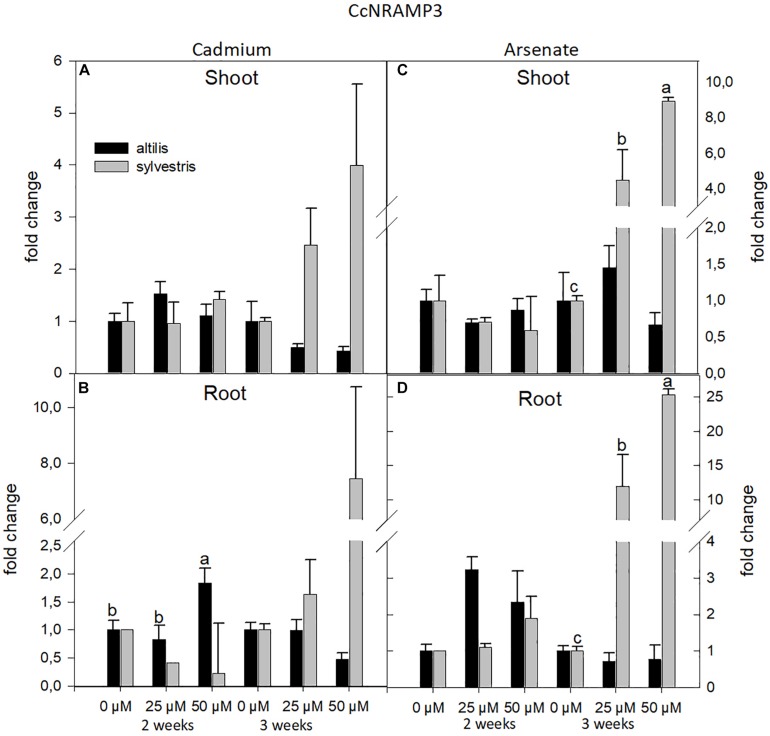
The level of gene expression of NRAMP3 in seedlings of *altili*s and *sylvestris* grown up to 2 and 3 weeks under Cd **(A,B)** and As **(C,D)** at different concentrations. The fold change is the Ct value with respect to the housekeeping genes (EF1α and GAPDH) that is considered 1. The error bars represent the error mean of three biological replicates. Letters indicate only significantly different values (*P* ≤ 0.05) between concentrations in each moment of sampling.

In both genotypes, *altilis* and *sylvestris*, transcriptional levels can be influenced by the concentration of metals in the medium, type of metals, and time of growth. As regards the NRAMP3 gene, its expression level was strongly influenced by the genotype and the time of growth (*P* < 0.05) (Figure 4). The ANOVA at one way showed a high relation between concentration of metals and gene expression, more in *sylvestris* than in *altilis* (*P* < 0.01). For both shoots and roots, NRAMP3 was expressed higher in *sylvestris* and down-regulated in *altilis*. In particular, after 3 weeks, at least a twofold and fourfold increase in expression levels was observed in *sylvestris*, respectively, with 25 and 50 μM regardless of the type of metal used.

**FIGURE 5:**
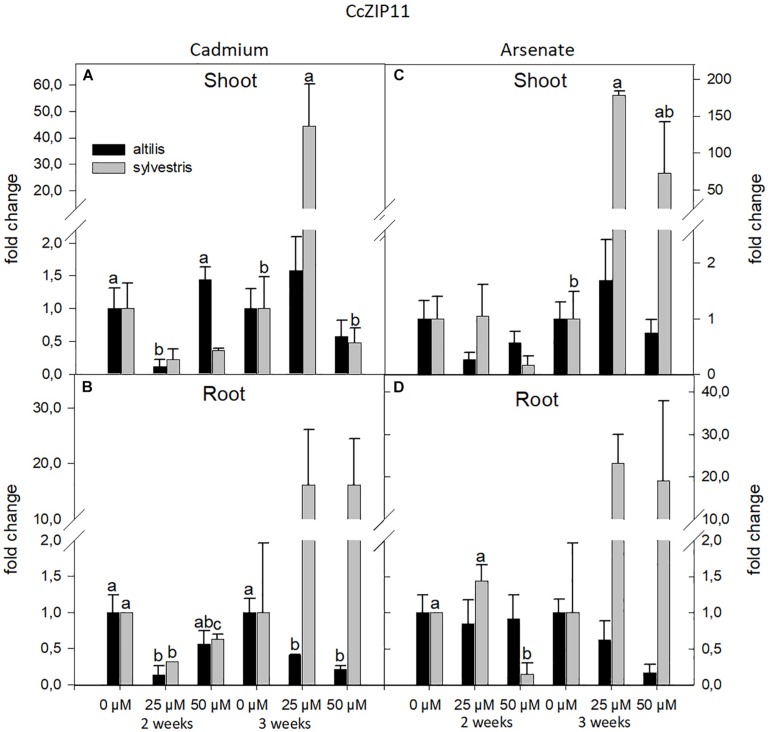
The level of gene expression of ZIP11 in seedlings of *altilis* and *sylvestris* grown up to 2 and 3 weeks under Cd **(A,B)** and As **(C,D)** at different concentrations. The fold change is the Ct value with respect to the housekeeping genes (EF1α and GAPDH) that is considered 1. The error bars represent the error mean of three biological replicates. Letters indicate only significantly different values (*P* ≤ 0.05) between concentrations in each moment of sampling.

Similarly to NRAMP3, the transcriptional levels of ZIP11 were influenced by the genotype and time of growth (*P* < 0.05). In both genotypes, the type of metal did not influence the transcriptional levels. In roots of *sylvestris* after 3 weeks, the level of ZIP11 mRNAs significantly increased in presence of Cd and As (*P* < 0.05), whereas in *altilis*, the transcriptional levels decreased with increasing levels of both metals (Figure 5).

**FIGURE 6:**
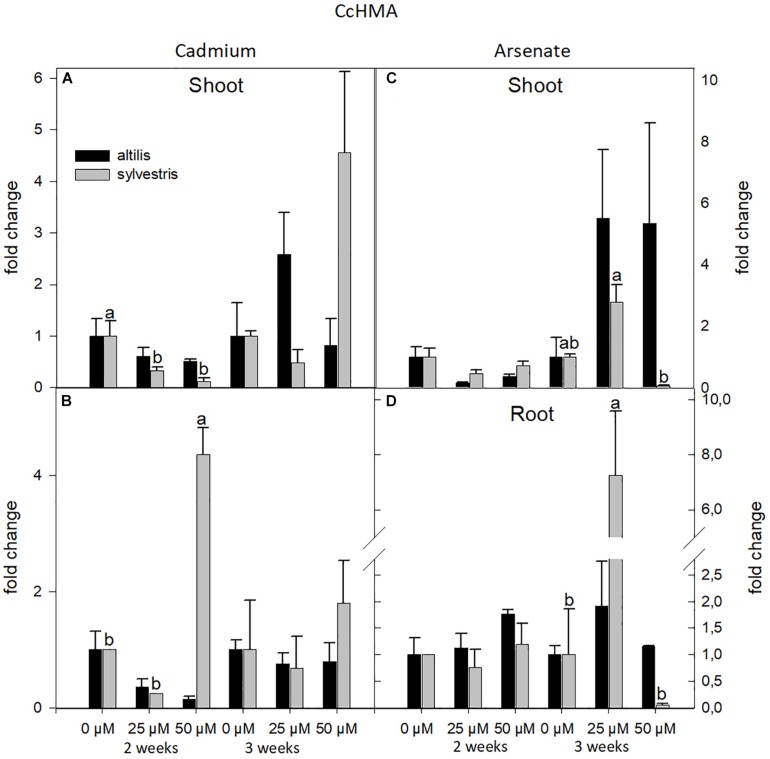
The level of gene expression of HMA in seedlings of *altilis* and *sylvestris* grown up to 2 and 3 weeks under Cd **(A,B)** and As **(C,D)** at different concentrations. The fold change is the Ct value with respect to the housekeeping genes (EF1α and GAPDH) that is considered 1. The error bars represent the error mean of three biological replicates. Letters indicate only significantly different values (*P* ≤ 0.05) between concentrations in each moment of sampling.

The expression of HMA was influenced by concentration and genotype. In particular, highly significant values, contributed by two of the three sources of variation (genotype, time, and concentration of metal), were observed in root treated with Cd (*P* < 0.05). Shoots and roots showed similar response to stresses, averaging the contribution of all the three sources of variation (*P* < 0.05), but the transcriptional level at 3 weeks in shoots of *altilis* with As was threefold increased than in *sylvestris* (Figure 6).

**FIGURE 7:**
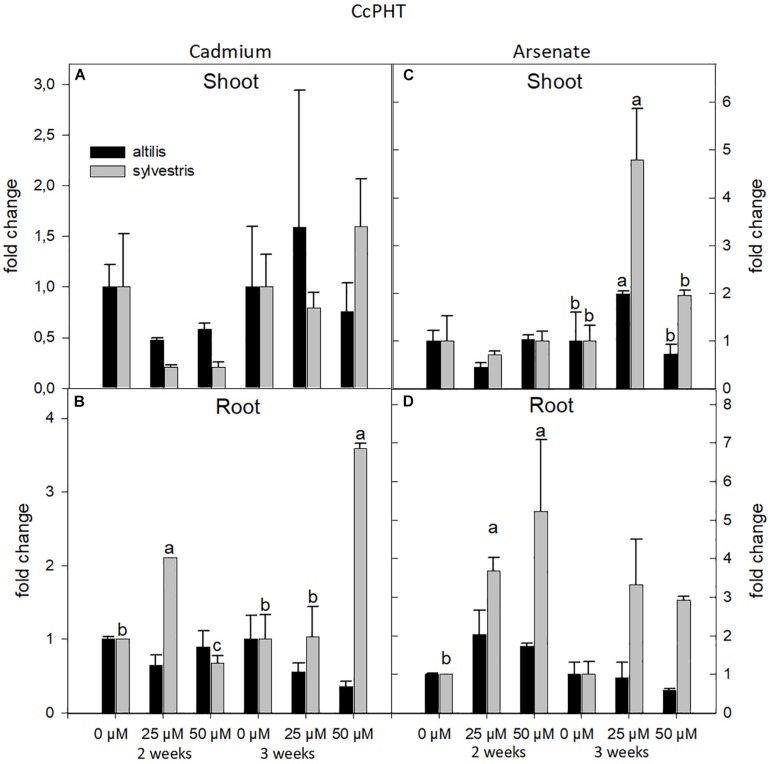
The level of gene expression of PHT in seedlings of *altilis* and *sylvestris* grown up to 2 and 3 weeks under Cd **(A,B)** and As **(C,D)** at different concentrations. The fold change is the Ct value with respect to the housekeeping genes (EF1α and GAPDH) that is considered 1. The error bars represent the error mean of three biological replicates. Letters indicate only significantly different values (*P* ≤ 0.05) between concentrations in each moment of sampling.

The transcriptional level of PHT was strongly influenced by the metal and genotype. In particular, highly significant values, contributed by the three sources of variation (genotype, time, and concentration of metal), were observed in shoot and root treated with As (*P* < 0.01). In roots, but not in shoots, the expression of the PHT transcript, averaging the contribution of all the three sources of variation, increased compared to control, and this was more evident in *sylvestris* than *altilis* (*P* < 0.01). The type of metal affected the expression of PHT. In fact, in presence of As, the PHT transcriptional level showed a twofold increase with respect to Cd. In particular, after 2 weeks in the *sylvestris* roots, the expression increased linearly with the concentration of metal ranging from zerofold, threefold, and to fivefold at 0, 25, and 50 μM of Cd, respectively (Figure 7). Under As treatment, the genotype (*sylvestris*) was the factor that more markedly contributed to transcriptional increase in both roots and shoots after 3 weeks of treatment (*P* < 0.001).

Moreover, the ABCC transcriptional levels were mainly influenced by the treatments used (type of metal and its concentration). In particular, highly significant values, contributed by the three sources of variation (genotype, time, and concentration of metal), were observed in root treated with As (*P* < 0.001).

**FIGURE 8:**
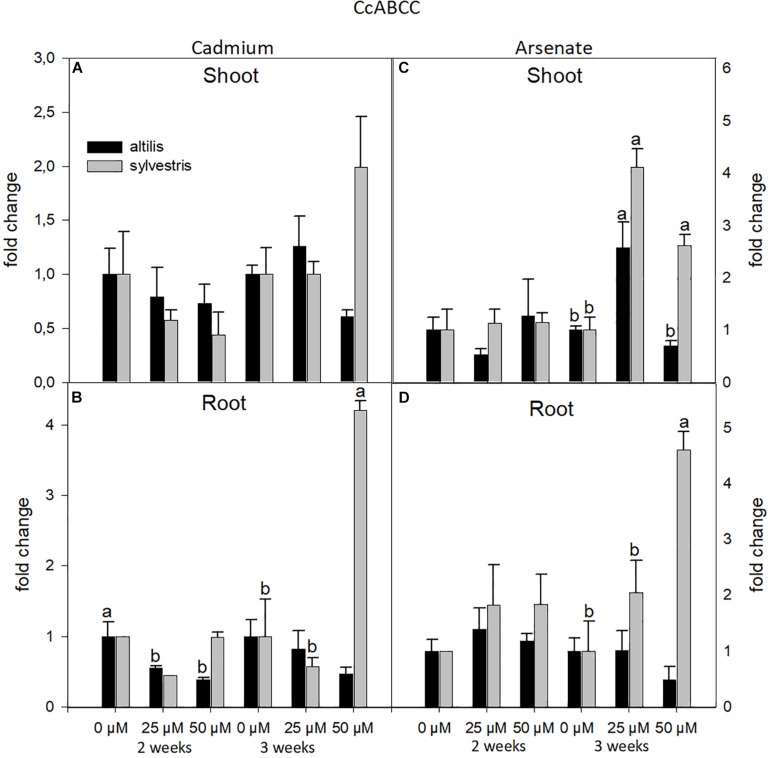
The level of gene expression of ABCC in seedlings of *altilis* and *sylvestris* grown up to 2 and 3 weeks under Cd **(A,B)** and As **(C,D)** at different concentrations. The fold change is the Ct value with respect to the housekeeping genes (EF1α and GAPDH) that is considered 1. The error bars represent the error mean of three biological replicates. Letters indicate only significantly different values (*P* ≤ 0.05) between concentrations in each moment of sampling.

On average, ABCC gene expression was not affected by the organ type (shoots or roots), but it was mainly influenced by the type of metal. In fact, with As, and not with Cd, the ABCC transcriptional levels increased by twofold in *sylvestris* compared to *altilis* (Figure 8). In particular, in wild cardoon, in shoots at 3 weeks, and in roots with As at 2 and 3 weeks, the ABCC mRNA was up-regulated, showing a clear involvement of this gene in As response (*P* < 0.01).

### Principal Component Analysis

**FIGURE 9:**
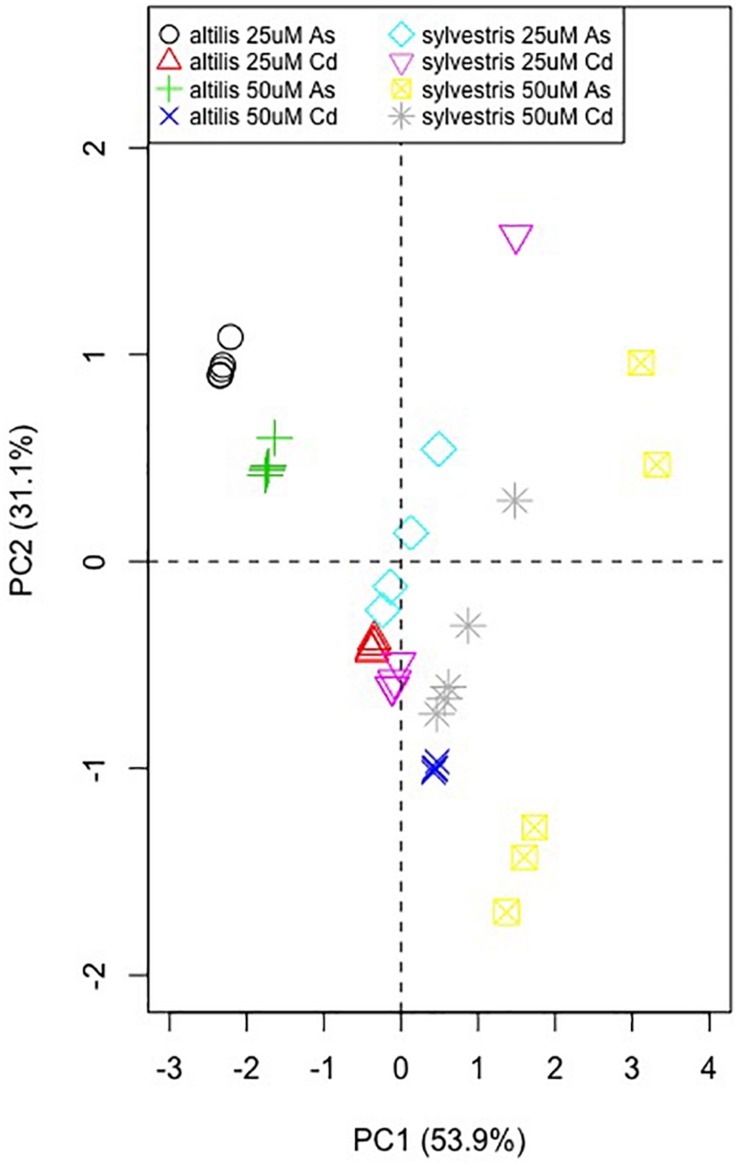
Principal component analysis using the RT-qPCR fold-change values of the five metal-response–associated genes along with the shoot and root length.

The PCA obtained using gene expression data and morphological characteristics in response to the presence of metals shows that for both varieties the values range from the first to the fourth quarter (Figure 9). However, *altilis* forms more compact clusters, which are more dispersed for sylvestris, instead. Moreover, the cluster localization is more influenced by metal type for *altilis*, whereas it is more dependent on the metal concentration for *sylvestris*.

## Discussion

In this study, we observed that salt stress did not reveal any significant effects on germination of domestic cardoon, while it linearly limited the development of fresh sprout biomass with the increase in NaCl concentrations. However, dry weight of sprouts did not show any significant variation between the salt and the control conditions, suggesting that the NaCl presence influences the water uptake capability of the plant decreasing water content of the seedlings. This salinity tolerance trait, in the early developmental stages, can be considered a plant adaptive strategy that allows us to consider *C. cardunculus* as a facultative halophyte species as reported by Benlloch-González et al. (2005).

As expected, salt stress induced the synthesis of phenolic substances in proportion to the increase in NaCl concentrations, confirming the important role of these molecules for the tolerance to stress conditions in plants. As well as for the total phenols, the trend of AA was directly proportional to salt concentration, demonstrating that the increase in the polyphenol content corresponds to a greater AA. These results reveal the possible use of salinity as an efficient technique for increasing the secondary metabolite content in plants grown for nutraceutical use, as documented for other elicitors or species (Bistgani et al., 2019; Hassini et al., 2019). In particular, in *C. cardunculus*, which is well-known for its high amount of polyphenolic compounds (Pandino et al., 2011), technical interventions based on the variation of NaCl concentrations in the germination solution could effectively modulate the bioactive molecules content in the sprouts.

In this study, for the first time, the influence of As and Cd on the growth of different genotypes of *C. cardunculus* sprouts was investigated. Their presence reduced the sprout growth in a significantly different way depending on the *C. cardunculus* genotypes. In particular, the *sylvestris* root and shoot lengths were mainly influenced by the concentration of metals; instead, the *altilis* growth was more affected by both the type of metal and its concentration. This finding is in accordance with growth rate documented for wheat and halophytes species in presence of heavy metals (Öncel et al., 2000; Leonardi et al., 2016; Amari et al., 2017).

Moreover, in the present study, we identified five *C. cardunculus* genes, NRAMP3, ZIP11, HMA, PHT, and ABCC, which seem to be involved in heavy metal stress response. Our results showed that in *sylvestris*, NRAMP3 expression is up-regulated in roots in presence of As or Cd with an increase in expression levels with longer treatments. This result agrees with Fallen et al. (2005), in which NRAMP3 and NRAMP4 were associated with Cd2^+^ efflux from the vacuole. Their overexpression increased Cd sensitivity and determined the release of vacuolar Fe2^+^ in *Arabidopsis*. Instead, no data are available in literature on NRAMP3 expression with respect to As.

ZIP proteins are generally responsible for the metal-ion homeostasis through the uptake of cations into the cytosol (Colangelo and Guerinot, 2006; Hou et al., 2017). Usually ZIP transporters are involved in the uptake and accumulation of Fe and Zn, but may also be responsible for Cd or other heavy metal transport (Guerinot, 2000). In *Solanum torvum* roots, IRT2 and ZIP11 are associated with Zn transport (Xu et al., 2012). In the present study, expression of the ZIP11 transporter in wild cardoon was higher in shoot and roots subjected to Cd treatment. Similarly, ZIP11 mRNA levels increased after 3 weeks of exposure of the seedlings to As, and this is the first study to document the expression variation of this gene in association with As presence.

The uptake of As(V) in plants occurs via inorganic phosphate (Pi) system, because Pi transporters cannot distinguish between the similar electrochemical profiles of Pi and As(V) (Sánchez-Pardo et al., 2015). In our experiments, in *sylvestris* genotype, the phosphate transporter was up-regulated in roots under As treatment, expression of which was strongly influenced by the concentrations of metal. In *altilis*, the expression levels of PHT were elevated also in control; probably for this reason, we did not observe any significant variation in the gene expression levels across the treatments.

These results can be associated with what was observed by Di Tusa et al. (2016), who showed in *Pteris vittata* an increase in As accumulation when the plants expressed PvPht1;3. In *Arabidopsis*, the expression pattern of PHT1;1 in the presence of As(V) decreased significantly as compared to limiting Pi condition in the natural variants, whereas the expression of PHT1;4 was higher in presence of As(V) to limiting Pi condition (Shukla et al., 2015).

Despite the documented role of the HMAs in the heavy metal transport in other species (Verbruggen et al., 2009; Fan et al., 2018), the identified cardoon HMA did not show a significant difference in the expression levels in the presence of Cd or As as measured for the other genes. Our results indicate that its expression level was mainly influenced by concentration and genotype, with the *altilis* showing to be more sensitive with respect to *sylvestris*.

In cardoon, ABCC transcriptional levels, measured under As treatment, in roots of *sylvestris*, remained up-regulated compared to untreated sample. The increase in the expression was influenced by the time of exposure, with the highest level at 50 μM after 3 weeks. A similar response was observed in shoots after 3 weeks of As treatment. These results are in accordance with Song et al. (2010), who showed that *Arabidopsis* isoforms AtABCC1 and AtABCC2 mediate AsIII–PC complex transport to the vacuole, and overexpression of AtABCC1 increases As tolerance only when coexpressed with phytochelatins (PCS). In rice, a similar ABC transporter, OsABCC1, is critical for the vacuolar AsIII–PC sequestration and As detoxification, thus reducing As accumulation in rice grains. For this reason, knockout of OsABCC1 leads to the increase in As sensitivity (Song et al., 2014).

The results obtained in the present study lead us to conclude that cardoon seed germination and seedling establishment can take place in salt and heavy metal stress conditions. In addition, we documented the possibility to use abiotic stresses to improve the bioactive molecules content in cardoon sprouts. Moreover, we identified some important genes encoding for metal transportation that may be involved in the uptake and translocation of As and Cd in *C. cardunculus*.

These findings, in a context of climate change and environmental pollution, can be useful tools for the possibility of exploiting marginal lands for the cultivation of species, such as cardoon plants, able to develop in stressed environments and suitable for green chemistry and energetic purposes. This chance could represent an economically valid alternative for farmers and for the agriculture of the future.

## Data Availability Statement

The raw data supporting the conclusions of this article will be made available by the authors, without undue reservation, to any qualified researcher.

## Author Contributions

All authors had full access to all of the data in the study and take responsibility for the integrity of the data and the accuracy of the data analysis, approved the final version of the manuscript. SR, HP conceived and designed the study. HP, CG, VT, and GP contributed to production and assembly of data. HP, CG, VT, GP, and SR analyzed and interpreted the data. HP, CG, VT, and GP drafted the manuscript. SR critically revised the manuscript for important intellectual content. HP and GP contributed to the statistical analysis. SR supervised the study.

## Conflict of Interest

The authors declare that the research was conducted in the absence of any commercial or financial relationships that could be construed as a potential conflict of interest.

## Footnotes

1 www.artichokegenome.unito.it
