# Supplementary material for: Cynara cardunculus L. as a Multipurpose Crop for Plant Secondary Metabolites Production in Marginal Stressed Lands
Source: Front Plant Sci. 2020 Mar 31;11:240. doi: 10.3389/fpls.2020.00240 (PMC7136453; doi:10.3389/fpls.2020.00240)
Supplement: Supplementary file 2 [file Table_2.docx]

**Supplemental Material:**

**Table S2.** Analysis of the obtained contigs with BLASTP against different portions of nucleotide collection (nr) database to confirm the presence of the protein domain.

| **Gene** | **BLASTP results using nr database excluding *Cynara cardunculus* (taxid: 4265)** | | | | | | **BLASTP results limiting the nr database to *Cynara cardunculus* (taxid: 4265)** | | | | |
| --- | --- | --- | --- | --- | --- | --- | --- | --- | --- | --- | --- |
|  | **GenBank sequence ID** | **Species name** | **Total Score** | **E-value** | **Identities** | **Protein domain detected** | **GenBank sequence ID** | **Total Score** | **E-value** | **Identities** | **Protein domain detected** |
| *NRAMP3* | XP_021992420.1 | *Helianthus annuus* | 712 | 0 | 91.21% | NRAMP | KVH92457.1 | 751 | 0 | 99.48% | NRAMP |
| *ZIP11* | PWA40765.1 | *Artemisia annua* | 542 | 0 | 70.00% | ZIP | XP_024967086.1 | 646 | 0 | 97.86% | ZIP |
| *HMA* | XP_021982455.1 | *Helianthus* *annuus* | 774 | 0 | 88.57% | ZntA | XP_024959995.1 | 854 | 0 | 99.52% | ZntA |
| *ABCC* | RWR88753.1 | *Cinnamomum micranthum* | 206 | 3E-61 | 65.27% | ABC transporter | KVH87904.1 | 394 | 1E-108 | 100.00% | ABC transporter |
| *PHT* | AGK29560.1 | *Chrysanthemum x morifolium* | 960 | 0 | 92.77% | Inorganic Phosphate Transporter | XP_024996153.1 | 1019 | 0 | 99.40% | Inorganic Phosphate Transporter |
| *GAPDH* | AGX26868.1 | *Saussurea involucrata* | 315 | 6E-106 | 97.42% | GAPDH-I | XP_0249903292.1 | 319 | 3E-110 | 98.71% | GAPDH-I |
| *EF1* | PLY82430.1 | *Lactuca sativa* | 836 | 0 | 98.77% | EF-1_alpha | XP_024968429.1 | 842 | 0 | 100.00% | EF-1_alpha |
